# Handgrip strength attenuates the adverse effects of overweight on cardiometabolic risk factors among collegiate students but not in individuals with higher fat levels

**DOI:** 10.1038/s41598-019-43471-5

**Published:** 2019-05-06

**Authors:** Antonio Garcia-Hermoso, Alejandra Tordecilla-Sanders, Jorge Enrique Correa-Bautista, Mark D. Peterson, Mikel Izquierdo, Daniel Prieto-Benavides, Carolina Sandoval-Cuellar, Katherine González-Ruíz, Robinson Ramírez-Vélez

**Affiliations:** 1 Laboratorio de Ciencias de la Actividad Física, el Deporte y la Salud, Facultad de Ciencias Médicas, Universidad de Santiago de Chile, USACH, Santiago, Chile; 2 CEMA Research Group, Rosario University, Cundinamarca Bogotá, Colombia; 3 Department of Health Sciences, Public University of Navarra, Navarrabiomed, IdiSNA, CIBER of Frailty and Healthy Aging (CIBERFES), Pamplona Navarra, Spain; 4 Department of Physical Medicine and Rehabilitation, University of Michigan, Ann Arbor, Michigan United States; 5 Grupo CORPS. Facultad de Ciencias de la Salud, Universidad de Boyacá, Tunja, Boyacá, Colombia; 6 Grupo de Ejercicio Físico y Deportes, Vicerrectoría de Investigaciones, Programa de Fisioterapia, Universidad Manuela Beltrán, Bogotá, Colombia; 7 Department of Health Sciences, Public University of Navarra, Navarrabiomed, IdiSNA, Pamplona Navarra, Spain

**Keywords:** Metabolic syndrome, Risk factors

## Abstract

The aims of this study are to (i) examine a clustered metabolic syndrome composite score (MetScore) and fatness among college students across body mass index (BMI) categories, and (ii) determine whether fit individuals have lower MetScores, fewer individual metabolic syndrome components, and lower fatness than unfit individuals across BMI categories. A total of 1,795 participants aged  >18 years who participated in The FUPRECOL Study were selected for the present analyses. Handgrip strength was tested by a grip dynamometer and used to classify adults as fit or unfit. Among all participants, MetScore, percentage of body fat, and visceral adiposity increased linearly across the BMI categories among college students (all P < 0.001). Individuals who were overweight and fit had a lower MetScore (−0.6 SD; P = 0.02), body fat percentage (−2.6%; P < 0.001) and visceral adiposity (−0.2; P = 0.01) than unfit peers. Moderately fit obese individuals had significantly lower visceral fat levels than unfit obese peers (−3.0; P = 0.03). These results suggest that having adequate handgrip strength-a proxy of overall strength capacity-may attenuate obesity-related cardiometabolic risk. Moreover, weight loss should be recommended to all individuals with obesity, even among those who are currently considered fit.

## Introduction

Several cross-sectional^1,2^ and prospective^3^ studies have provided evidence suggesting that muscular strength is protective against cardiometabolic risk factors. Specifically, in a follow-up study of young adults of 20 years, Fraser *et al*.^3^ showed that being in the lowest tertile of muscular fitness was associated with the risk of metabolic syndrome. Modifiable risk factors, including muscular strength and a healthy diet, protect youth against cardiometabolic risk factors^4^.

A clinically feasible measure of muscle fitness is the handgrip strength test, which can predict declines in physical and mental capacities among older adults^5^ and even mortality^6^. Among youth^7,8^, young adults^9^, and adults^10^, higher muscular strength capacities are also associated with better cardiometabolic risk profiles. For example, Vaara *et al*.^11^ demonstrated that greater muscular strength was inversely associated with C-reactive protein and interleukin-6 concentrations in young adult men, independent of aerobic fitness. Limitations of previous studies examining the link between muscular strength and cardiometabolic risk factors in youth and collegiate students include small sample sizes^12^, including only populations with low/middle socioeconomic status^13^, failure to include clinically relevant endpoints such as insulin resistance or inflammatory markers^14^, and failure to adjust for relevant confounders or covariates^15^.

High adiposity levels and muscular weakness are important modifiable risk factors for cardiovascular disease^9^. Indeed, the fat-but-fit paradox suggests that individuals that are obese and yet have moderate to high fitness levels may not actually have an increased risk of mortality than normal-weight unfit adults^16^. A cross-sectional study of collegiate students suggested that this paradox is not necessarily supported^9^. Although scientific evidence exists regarding the role of muscular fitness preservation in preventing cardiometabolic risk factors in several populations, there has been no research to establish minimum strength capacity levels to predict risk of metabolic syndrome among Latin American college students. It is noteworthy that the majority of studies in this age group were stratified by the median for both fitness and fatness categories and not by standardized cutoff values. Recently, our research group determined cutoff values for normalized grip strength (NGS) in a large collegiate student population from Colombia, for the detection of metabolic syndrome^17^.

Given the equivocal evidence regarding the link between muscular strength, fatness, and risk of metabolic syndrome in college-aged students, the aims of the study were (i) to examine a clustered MetScore and fatness among college students across BMI categories, and (ii) to determine whether fit individuals have lower MetScores, fewer individual metabolic syndrome components, and lower fatness than unfit individuals across BMI categories.

## Materials and Methods

The present study evaluated data from The FUPRECOL study, which is a no-representative survey conducted between 2014 and 2017 on 716 men and 1,126 women from Colombia (*n* = 1,842). The design of the study and data collection has previously been described in detail^18^. We removed participants due to missing (*n* = 20, 1,1%) or erroneous data entries (*n* = 15, 0.8%), and those without a recorded age or valid date of birth (*n* = 12, 0.6%). Ultimately, 1,795 volunteers (61.5% women) were selected for inclusion in the present.

Participants who had clinical diagnosis of a major systemic disease, including conditions such as cancer, systemic lupus erythematosus, diabetes mellitus, chronic inflammatory conditions such as rheumatoid arthritis, hypothyroidism or hyperthyroidism, multiple sclerosis, and infectious conditions, were excluded from the analyses. No compensation was provided for participation in this study. Signed informed consent was obtained from all FUPRECOL Study participants. The protocol was in accordance with the Declaration of Helsinki (World Medical Association for Human Subjects) and its later amendments. All procedures have been approved by the ethics committee of the UMB (Code N° 01-1802-2013).

Body mass (kg) was measured with an electric scale (Model Tanita^®^ BC-420^®^, Tokyo, Japan). Standing height was assessed with a stadiometer (Seca^®^ 274, Hamburg, Germany). Body mass index (BMI) was calculated as body mass (kg)/height (m^2^), and BMI status was categorized according to the World Health Organization criteria^19^. Waist circumference (WC) was measured midway between the lowest rib and the top of the iliac crest after gentle expiration with a non-elastic flexible tape measure (Lufkin W606PM^®^, Parsippany, NJ, USA), as recommended by the ISAK guidelines^20^. Physical examination was carried out by the same investigators specifically trained. The technical error of measurement values was less than 2% for all anthropometric variables.

The percentage of body fat and the visceral fat score/levels were determined by bioelectrical impedance analysis (BIA) (Tanita BC 420 MA/SC-331S®, Tokyo, Japan). The BIA Monitor (Model BC 420 MA/SC-331S®) provides a visceral fat rating from 1–59. The reliability coefficient was significant for the percentage of body fat, fat mass, fat-free mass and muscle mass (intraclass correlation coefficient = 0.95, P < 0.001, intra-observer technical error [% reliability] of the measurements = 95%, and inter-observer technical error of measurement = 0.55). In a study to validate 2 portable BIA devices; for body composition assessment multiple regression analysis showed clinically acceptable agreement between the Tanita BC 420 MA^®^ and SC-331S^®^ device and magnetic resonance imaging for visceral adipose tissue and fat mass measurements (R^2^ > 0.70, r > 0.84)^21^. A detailed description of the BIA technique can be found in a previous study^22^.

Blood pressure (BP) was measured from the right arm with an Omron® HEM 705 CP (Omron® Healthcare Europe B.V., Hoofddorp, Netherlands), with participants sitting still using, an automatic oscillometric sphygmomanometer with a cuff of 107 × 63 mm in size, following the recommendations of the European Heart Society^23^, after 5 min rest. Mean arterial blood pressure (MAP) was calculated as MAP = diastolic BP + (0.333 * [systolic BP x 2 diastolic BP]).

Blood samples were drawn in the morning after 10–12 hours of fasting, between 07:00 and 09:00 a.m. Capillary blood samples (40 µL) were collected to determine serum biochemical parameters, including of glycaemia, high-density lipoprotein cholesterol (HDL-C), triglycerides (TG), and total cholesterol (TC), using Cardiocheck^®^ equipment (Mexglobal SA, Parsippany, NJ, USA). Low-density lipoprotein-cholesterol (LDL-C) was measured using Friedewald’s Formula if triglyceride values were ≤ 400 mg/dL.

A cluster MetScore was constructed as the sum of the Z-scores, based on age and sex from the following equation according to the International Diabetes Federation criteria cutoff values^24^: MetScore = ((WC – ♂90 or ♀80)/SD) + ((MAP – 100)/SD) + ((♂40 or ♀50 – HDL-C)/SD × (−1)) + ((TG – 150)/SD) + ((glucose – 100)/SD). Individuals with a cardiometabolic risk score + 1 SD above the mean were identified as having increased cardiometabolic risk and a lower MetScore being indicative of a healthier risk profile.

Grip strength was tested by a digital dynamometer (T.K.K. 5401, Grip-D Smedley, Takei, Japan), with adjustment for the hand size of each collegiate students. The participants stood upright with their feet roughly shoulder-width apart. Their arms were allowed to fall obliquely with the palms facing inward. Each individual squeezed the dynamometer twice, as hard as they could alternating hands. The best score for each hand was recorded in kilograms (kg), accurate to one decimal place^22^, and the score (kg) was calculated as the average of the scores for the left and right hands. Inter-rater reliability was assessed by determining the intraclass correlation coefficient (0.98, CI 95% 0.97–0.99).

Because there is substantial covariance between upper muscular fitness and body mass and because the link between strength and both chronic health and physical function is directly mediated by the proportion of strength relative to body mass, grip strength was normalized as handgrip strength (NGS) per body mass, i.e., (handgrip strength in kg)/(body mass in kg). In males, “unfit” and “fit” NGS values at these points were <0.47 and ≥0.48, respectively. In females, these cutoff values were <0.33 and ≥0.34, respectively^17^. These cutoff values are associated with the detection of metabolic syndrome in Colombian college students^17^. To test the “fat but fit” paradox, we divided the eligible students into four categories: normal-weight and fit, normal-weight and unfit, obese and fit, and obese and unfit.

The “FANTASTIC” validated questionnaire was used to collect comprehensive information about lifestyle via a personal interview with college students (family, physical activity [PA], nutrition, tobacco toxins, alcohol, sleep/stress, personality type, insight, career)^25^. Finally, individuals completed a questionnaire regarding medical history, including personal and family history of cardiovascular disease (CVD).

Data are presented as mean values, standard deviations, and percentages. Statistical normality was tested using both statistical (Kolmogorov–Smirnov test) and graphical procedures (normal probability plots). Linear regression analyses with a cluster MetScore, percentage of body fat, and visceral adiposity as the dependent variable and BMI category as the independent variable were used to assess trends. Means of MetScore and fatness markers were calculated for each BMI category and compared using ANCOVA tests and Bonferroni pairwise comparisons. Differences in MetScore, each cardiometabolic risk factor, and fatness markers (percentage of body fat and visceral fat rating) across each BMI category (i.e., underweight, normal-weight, overweight, moderate obesity, and severe obesity) and handgrip strength category (unfit vs fit) were analyzed using a one-way ANCOVA. All analyses were adjusted for age, sex, tobacco and alcohol intake, and self-report PA levels. Finally, a linear regression analysis was used to determine the independent relationship between handgrip strength and MetScore, controlling for the abovementioned variables and percentage of body fat or visceral fat. All analyses were adjusted for age, sex, tobacco and alcohol use, and PA levels. Data were entered using the Statistical Package for Social Sciences (SPSS) software, version 24^®^ (IBM Corp., Armonk, N.Y., USA). Statistical significance was described as P < 0.05.

## Results

The anthropometric, fatness markers, handgrip strength and individual cardiometabolic risk factors assessed in this study are shown according to the categories of BMI in Table 1.

**Table 1:** Means and SDs for the anthropometric, fatness, muscle strength and cardiometabolic risk factors stratified by BMI categories*(n = 1,795).

Characteristic	Underweight (n = 110)	Normal-weight (n = 1,212)	Overweight (n = 379)	Moderate obesity (n = 82)	Severe obesity (n = 14)
Age (years)	20.1 ± 1.3	20.3 ± 2.0	21.1 ± 1.9	21.9 ± 1.5	21.9 ± 1.8
WC (cm)	63.9 ± 5.4	71.4 ± 6.1	81.2 ± 6.8	91.7 ± 9.1	101.7 ± 17.2
Body mass index (kg/m^2^)	17.1 ± 1.0	21.8 ± 1.8	26.8 ± 1.4	31.8 ± 1.4	38.7 ± 3.8
Body fat (%)	12.5 ± 6.2	20.4 ± 7.2	29.3 ± 6.9	36.2 ± 7.0	39.4 ± 9.2
Visceral fat rating	1.2 ± 0.5	1.4 ± 0.9	4.1 ± 1.6	7.4 ± 2.5	10.6 ± 6.3
Handgrip (kg)	26.4 ± 7.2	29.9 ± 9.4	31.0 ± 9.6	31.9 ± 10.0	32.5 ± 11.1
Normalized grip strength	0.55 ± 0.13	0.50 ± 0.12	0.43 ± 0.10	0.36 ± 0.09	0.31 ± 0.08
Systolic blood pressure (mmHg)	109.4 ± 12.4	113.4 ± 12.1	118.2 ± 12.7	124.8 ± 11.7	124.8 ± 9.5
Diastolic blood pressure (mmHg)	72.5 ± 12.6	71.7 ± 9.6	74.8 ± 11.1	77.9 ± 9.5	78.3 ± 6.1
Mean arterial pressure (mmHg)	91.0 ± 10.7	92.5 ± 9.6	96.5 ± 10.4	101.4 ± 8.9	101.5 ± 5.2
Total cholesterol (mg/dL)	134.7 ± 26.9	140.3 ± 33.4	144.7 ± 32.6	146.5 ± 32.5	136.8 ± 28.7
Triglycerides (mg/dL)	79.5 ± 37.6	84.3 ± 40.4	106.5 ± 53.2	120.2 ± 73.6	123.5 ± 49.9
LDL-C (mg/dL)	79.6 ± 21.8	84.9 ± 26.6	88.2 ± 26.6	87.4 ± 26.1	77.5 ± 20.8
HDL-C (mg/dL)	44.9 ± 11.9	43.6 ± 12.1	38.6 ± 12.2	36.4 ± 8.2	44.9 ± 11.9
Glycaemia (mg/dL)	84.3 ± 10.8	84.6 ± 12.0	87.8 ± 10.8	89.2 ± 10.5	94.4 ± 11.6
MetScore	−5.86 ± 2.13	−4.68 ± 2.18	−1.97 ± 2.51	0.35 ± 2.65	2.40 ± 3.60
Tobacco (1 to 10 cigarettes per day), n (%)	17 (15.5)	129 (10.6)	42 (11.1)	9 (11.0)	2 (16.7)
Alcohol (1 to 7 times per week), n (%)	0 (0.0)	53 (4.4)	11 (2.9)	1 (1.2)	0 (0.0)
PA (5 times a week for >30 min), n (%)	40 (36.4)	362 (29.9)	99 (26.1)	26 (31.7)	3 (25.0)

*BMI categories were computed using the cut points established by the (WHO) criteria^19^. WC, waist circumference, PA, physical activity.

Table 2 shows the differences in cardiometabolic risk factors across the different BMI categories by handgrip strength categories (unfit and fit). Unfit normal-weight individuals showed high levels of TG (P = 0.039) and low levels of HDL-C (P = 0.008) compared to fit peers. Additionally, unfit moderately obese collegiate students had higher levels of LDL-C than fit counterparts (P = 0.025).

**Table 2:** Differences in each individual cardiometabolic risk factors through the different BMI categories by muscular strength categories (fit and unfit).

	Underweight (n = 110)	Normal-weight (n = 1,212)	Overweight (n = 379)	Moderate obesity (n = 82)	Severe obesity (n = 14)
**Mean arterial pressure (mmHg)**					
Fit	91.1 ± 10.8	92.5 ± 9.6	96.7 ± 10.4	100.9 ± 7.3	103.8 ± 4.6
Unfit	87.7 ± 4.1	92.8 ± 9.7	96.0 ± 10.5	101.7 ± 9.9	101.1 ± 5.4
*P*	0.591	0.748	0.553	0.692	0.534
**Triglycerides (mg/dL)**					
Fit	79.6 ± 37.9	83.5 ± 39.0	106.1 ± 53.5	118.9 ± 79.3	135.5 ± 41.7
Unfit	75.3 ± 31.8	91.4 ± 50.4	107.1 ± 52.5	121.1 ± 70.6	121.3 ± 52.8
*P*	0.848	0.039	0.882	0.898	0.728
**LDL-C (mg/dL)**					
Fit	79.8 ± 21.7	85.3 ± 26.6	87.2 ± 26.3	78.8 ± 21.0	72.5 ± 7.8
Unfit	74.5 ± 34.6	81.8 ± 26.0	91.3 ± 27.4	92.7 ± 27.7	78.5 ± 22.7
*P*	0.740	0.224	0.206	0.025	0.728
**HDL-C (mg/dL)**					
Fit	45.1 ± 12.0	43.9 ± 12.1	38.7 ± 12.1	37.9 ± 8.7	29.5 ± 0.7
Unfit	38.0 ± 8.7	40.9 ± 11.4	38.5 ± 12.5	35.5 ± 7.9	37.5 ± 17.5
*P*	0.310	0.008	0.871	0.185	0.548
**Glycaemia (mg/dL)**					
Fit	84.2 ± 10.9	84.5 12.0	87.3 ± 11.1	90.0 ± 10.5	101.5 ± 2.1
Unfit	86.3 ± 9.5	85.8 ± 12.2	89.4 ± 9.8	88.7 ± 10.6	93.1 ± 12.2
*P*	0.737	0.247	0.106	0.568	0.367

Analysis adjusted for age, sex, tobacco, alcohol, and physical activity met (5 times a week for >30 min).

**Figure 1:**
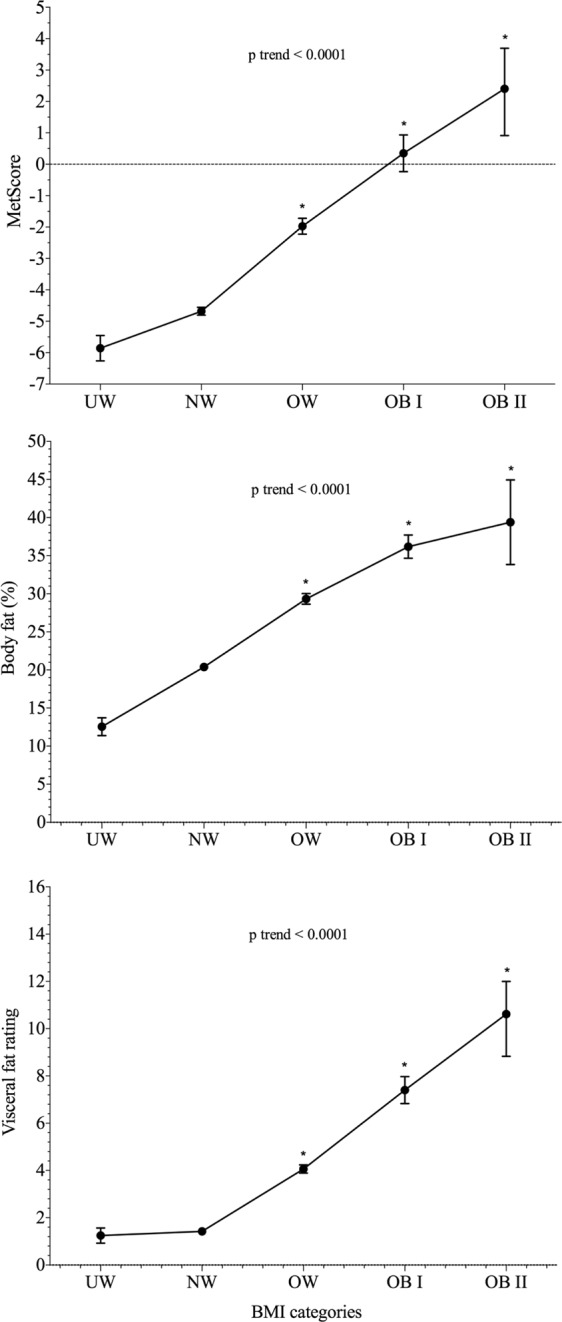
Differences in MetScore and fatness markers across BMI categories (n = 1,795). Data represents adjusted means from ANCOVA models and 95% error bars, after adjustment for age, sex, tobacco and alcohol use, and physical activity levels. UW, underweight; NW, normal-weight; OW, overweight; OB I, moderate obesity; and OB II, severe obesity. *P < 0.005 compared with normal-weight collegiate students.

Figure 1 shows the differences in MetScore and fatness markers across the different BMI categories. When using regression analysis, each increment in BMI category was associated with a 2.3**−**SD higher MetScore (β = 0.554, P trend < 0.001). Compared with normal-weight collegiate students, participants who were overweight, moderately obese or severely obese had 3.8**−**, 6.2**−**, and 8.2**−**SD higher MetScores, respectively. There were significant differences in MetScore between each BMI category (pairwise comparison range: P < 0.001–0.004). Similar linear associations of greater MetScore with higher BMI categories were also observed for percentage of body fat (β = 0.602, P < 0.001) and visceral fat (β = 0.750, P trend < 0.001). Compared with normal-weight individuals, underweight participants had a lower overall risk; however, the difference was only significant for the percentage of body fat (P < 0.001).

**Figure 2:**
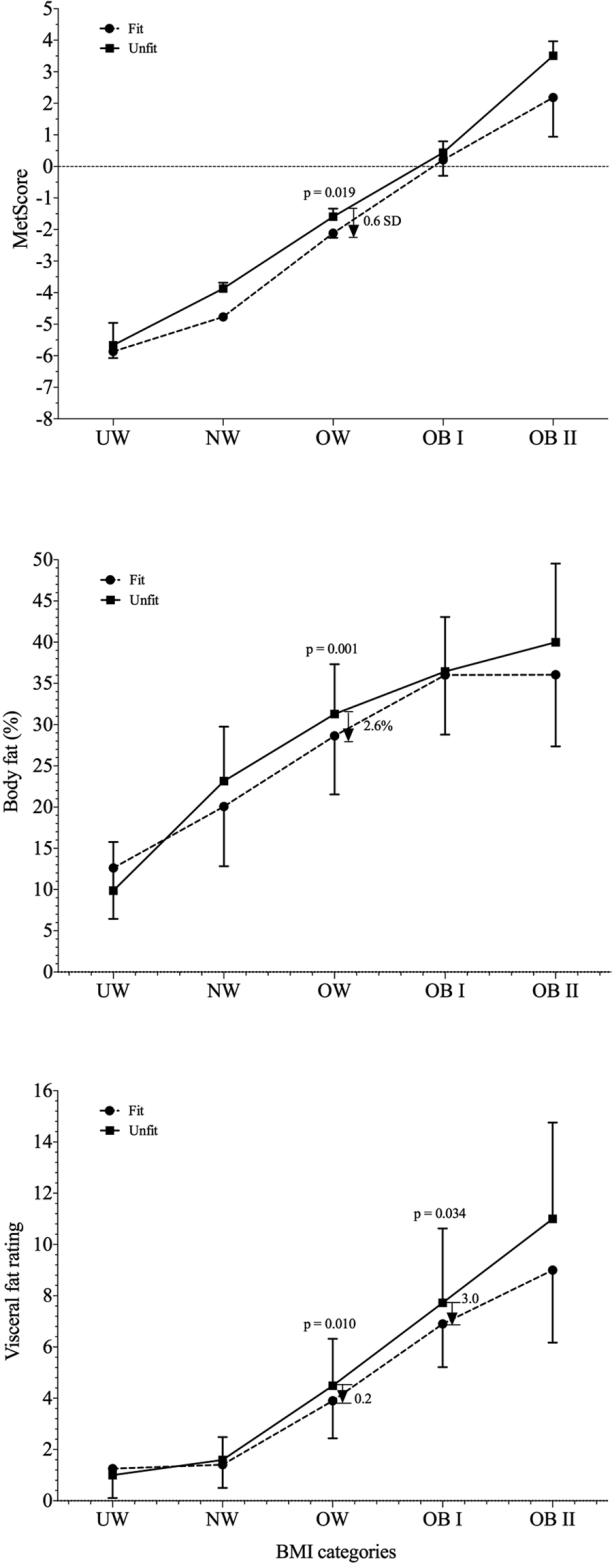
MetScore and fatness markers across BMI categories by fit and unfit individuals. The dashed line represents a value of zero for the scores, and a higher score represents a greater cardiometabolic risk. The arrow shows the reduction in the MetScore (expressed in the number of SDs), body fat (%) or visceral fat rating for the fit collegiate students compared with the unfit collegiate students in the OW or OB II group. UW, underweight; NW, normal-weight; OW, overweight; OB I, moderate obesity; and OB II, severe obesity.

Figure 2 displays the influence of handgrip strength on MetScore, body fat percentage and visceral fat among the different BMI categories. Overall, there was a trend showing that higher muscle strength was associated with lower MetScore (0.6 SD), percentage of body fat (2.6%) and visceral fat rating (0.2), particularly among overweight collegiate students (P < 0.05). Additionally, fit moderately obese collegiate students had significantly lower visceral fat rating levels than unfit peers (3.0; P = 0.03).

Finally, multiple regression analysis revealed that the NGS was negatively correlated with MetScore (ß = **−**0.484; P < 0.001); however, after including the percentage of body fat (ß = **−**0.159; P < 0.001) or visceral fat (ß = **−**0.204; P < 0.001), this association was attenuated.

## Discussion

The present study suggests that MetScore and fatness markers, specifically body fat and visceral adiposity, increase linearly across the BMI categories among collegiate students. Additionally, higher handgrip strength is associated with lower MetScore, body and visceral fat in overweight individuals, indicating a protective effect of muscular strength was present in individuals with these phenotypes but not in counterparts with higher fat levels. However, the limited number of subjects in the moderate and severe obesity categories requires a cautious interpretation of these results.

A recent report published by the American Heart Association^26^ indicates that obesity has a negative influence on cardiometabolic risk factors such as dyslipidemia, hypertension, glucose intolerance, and inflammatory markers; other authors have also indicated that severe obesity is associated with an increased risk of cardiovascular disease^27^. The Multi-Ethnic Study of Atherosclerosis study also suggested that greater obesity severity was associated with a higher MetScore and its components^28^. Similarly, the HERMEX study indicates that the severity of obesity is associated with adverse cardiometabolic risk factors in women and men^29^. Therefore, our results and those of previous studies in adults demonstrate that cardiometabolic risk and body composition increase with BMI status^28–30^. Both weakness and excess adiposity have been recognized as important risk factors associated with metabolic syndrome in young adults^31^ however, few studies have analyzed their combined effect on metabolic syndrome in college-aged individuals^9,32^. Our research group recently revealed that low-fat and fit young adults had better metabolic profiles compared to unfit and high-fat counterparts, clearly highlighting that the combination of unfit and high fat poses a substantial health hazard^9^. The Sacheck *et al*.^32^ study showed that high levels of muscular fitness in the higher fatness group were associated with attenuated continuous cardiometabolic risk score. Therefore, being physically fit can confer an added benefit to maintaining a healthy body composition. Moreover, another cross-sectional study among Korean young adults revealed that obese and unfit subjects had a higher risk of metabolic syndrome^33^ These studies collectively suggest that improvement of muscular fitness and reduction of fat are both important factors for the prevention of metabolic syndrome. In contrast, in the present study, handgrip strength seemed to attenuate the MetScore in only overweight individuals, indicating a protective effect of muscular strength was present in this phenotypes. Additionally, the overall results suggested that body composition attenuated the relationship between NGS and MetScore. Therefore, although evidence has shown that young fit adults with excess weight have lower metabolic risk than their unfit peers with excess weight^9,33^, our results do not fully support or completely refute the “fat but fit” paradox.

The significantly lower MetScore in overweight fit collegiate students compared with obese unfit peers in our study is in line with a previous study evaluating metabolic syndrome prevalence^34^, although the evidence is conflicting. Kim *et al*.^34^ demonstrated that excessive fatness and aerobic capacity are cumulatively associated with a higher prevalence of metabolic syndrome in overweight and obese adults. Therefore, this study demonstrated that aerobic capacity could modify the association between central obesity and the prevalence of metabolic syndrome. One possible mechanism linking muscular strength and favorable health is through reducing chronic low-grade inflammation^35^. For example, a study using data from the 1999–2002 NHANES demonstrated that muscular strength of the knee extensors may help attenuate systemic inflammation among those who are overweight, but may not be sufficient to reduce inflammation levels to those of normal-weight unfit individuals^36^. In contrast, the FATCOR study suggested that cardiorespiratory fitness is not associated with a lower burden of hypertension, metabolic syndrome and diabetes in the overweight and obese population^37^.

To the best of our knowledge, no studies have analyzed the “fat but fit” paradox using body fat and visceral parameters as dependent variables. Scientific evidence has determined that excessive visceral fat is one of the strongest contributing factors leaning to adverse cardiometabolic profiles that are associated with being obese^38^. A recent study suggested that fat mass and muscle mass are important measurements of nutritional status and extended the analysis of their impact on health outcomes in all BMI categories^30^. In the study mentioned above, Kim *et al*.^34^ also revealed that adults who have higher visceral adiposity, but also exhibited high fitness levels were at higher odds of developing metabolic syndrome than subjects with high visceral fat and low fitness levels. Additionally, our research group reported that the influence of muscular strength on MetScore was at least partially mediated by fatness, regardless of the fatness parameters used^9^. Likewise, in the present study, adequate handgrip strength was potentially protective against high visceral fat in overweight collegiate students but not in obese subjects. These findings indicate that weight loss should be recommended for all individuals with obesity^30^, including those who are fit.

Two strengths of this investigation were the inclusion of a large sample of college-aged students and the use of a clinically-feasible method for measuring muscle strength. However, as with all studies, there are some limitations that should be highlighted. First, due to the cross-sectional design of the study design, we were unable to draw conclusions about the causal direction of association between grip strength and cardiometabolic disease risk. Additionally, because we included otherwise healthy collegiate students, the generalization of our results to other populations may be limited.

## Conclusion

Our findings suggest that MetScore, body fat and visceral adiposity increase across BMI categories among collegiate students. Additionally, collegiate students who were overweight and had higher strength, as determined from NGS, had lower MetScores and levels of fatness markers than unfit peers. Our results indicate that higher handgrip strength relative to body mass may help attenuate cardiometabolic risk, and that weight loss and exercise should be recommended to all individuals with obesity, including those who are currently considered fit.

## Data Availability

The datasets generated during the current study are not publicly available due to the confidential nature of the material but are available from the corresponding author on reasonable request.
